# Effective Functional Pattern Between Prefrontal fNIRS Activity and Cognitive Performance in Patients With Parkinson's Disease: A Data‐Driven PLS Approach

**DOI:** 10.1002/brb3.70915

**Published:** 2025-09-25

**Authors:** Xinxin Chen, Bingjie Tian, Bingjie Ma, Shizheng Zhang, Xiangdong Huang, Jianping Huang, Zhenguo Liu, Miaomiao Hou

**Affiliations:** ^1^ Department of Neurology Wenzhou Central Hospital Wenzhou Zhejiang China; ^2^ WenZhou Panvascular Disease Management Center, The Wenzhou Central Hospital Wenzhou Zhejiang China; ^3^ Department of Nursing Huashan Hospital Fudan University Shanghai China; ^4^ Department of Painology Xinhua Hospital affiliated to Shanghai Jiao Tong University School of Medicine Shanghai China; ^5^ Department of Neurology Xinhua Hospital Affiliated to Shanghai Jiao Tong University School of Medicine Shanghai China

**Keywords:** brain‐behavior relationship, effective connectivity, functional near‐infrared spectroscopy, Parkinson's disease

## Abstract

**Background:**

Current clinical assessments often fail to detect early prefrontal dysfunction in Parkinson's disease (PD), highlighting the need for objective neuroimaging biomarkers to quantify causal connectivity patterns for early impairment detection.

**Methods:**

We enrolled 91 PD patients (71.08 ± 5.53 years) who underwent functional near‐infrared spectroscopy (fNIRS) recording during both resting‐state and verbal fluency task (VFT). Transfer entropy (TE) analysis was performed to quantify effective connectivity (EC) between prefrontal regions. Partial least squares (PLS) was then applied to examine multivariate relationships between task design, MoCA subscales, and VFT performance (verbal output).

**Results:**

PLS analysis revealed distinct EC patterns associated with task‐states (resting state vs. VFT) and cognitive performance. Specifically, EC within the prefrontal network was significantly enhanced during VFT compared to the resting state. These PLS‐derived patterns showed consistency with conventional paired *t*‐test results, reinforcing the robustness of findings. Stronger top‐down connectivity to the FPA‐R (the right frontopolar area) in the resting state was correlated with better language performance on the MoCA. Conversely, enhanced top‐down connectivity to the FPA‐L (the left frontopolar area) during VFT was correlated with poorer MoCA subscales in attention, abstraction, delayed recall, and orientation, as well as with reduced verbal output.

**Conclusion:**

PD patients with poorer cognitive performance exhibited enhanced top‐down modulation of the FPA‐L by other prefrontal regions, suggesting compensatory network adaptation. These findings identify FPA‐L as a critical hub for cognitive reserve in PD.

## Graphical abstract

This study integrated transfer entropy and partial least squares analysis of fNIRS data to quantify effective connectivity in 91 Parkinson's patients. We identified left frontopolar area as a crucial top‐down hub whose hyperconnectivity patterns correlated with specific cognitive deficits and may represent a key neural compensatory mechanism in PD.

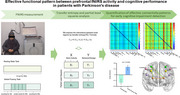

## 1 Introduction

Parkinson's disease (PD), the second most prevalent neurodegenerative disorder, presents with both motor and non‐motor symptoms. Among these, cognitive impairment stands out as a significant non‐motor symptom, affecting over 50% of patients and progressively worsening with disease progression (Thomas et al. 2020). In the early stages of the disease, cognitive decline primarily involves executive dysfunction, attentional deficits, and working memory impairment, while advanced stages exhibit broader dementia features associated with “subcortical dementia” and “cortical dementia” (Collins and Williams‐Gray 2016). Given this trajectory, early detection and timely intervention are critical for preserving social functioning and quality of life for individuals affected by Parkinson's disease dementia (PDD).

Growing evidence implicated the prefrontal cortex as a key neural substrate for cognitive decline in PD. Postmortem molecular studies (Zhu et al. 2024) and animal models (Tang et al. 2021; Haber and Behrens 2014) demonstrate its selective vulnerability, while neuroimaging reveals disrupted prefrontal‐striatal networks in PD patients (Rodriguez‐Rojas et al. 2022; Plantinga et al. 2018). Although dopaminergic deficiency remains central (Schrag et al. 2017), emerging evidence implicates α‐synuclein (Endo et al. 2024), neuroinflammation (Kouli et al. 2024), and tau pathology (Horvath et al. 2013). Notably, prefrontal dysfunction appears early in PD progression, making it a prime target for detecting initial cognitive impairment.

Cognitive decline in PD exhibits marked heterogeneity in progression patterns, necessitating objective neuroimaging biomarkers that can complement and enhance the interpretation of clinical cognitive scales (e.g., Montreal Cognitive Assessment, MoCA, and Mini‐Mental State Examination, MMSE). However, these scales lack sensitivity to early prefrontal network dysfunction—a key driver of cognitive impairment in PD (Aarsland et al. 2017). To address this gap, we conducted verbal fluency tasks (VFT), a well‐established prefrontal function paradigm, monitored by functional near‐infrared spectroscopy (fNIRS), which combines high temporal resolution with practical advantages for clinical deployment (Bonilauri et al. 2020). Notably, conventional assessments fail to characterize directional information flow within prefrontal circuits. To overcome this, we applied transfer entropy (TE) to quantify the effective connectivity relationship between prefrontal regions (Shu et al. 2024). This approach reveals how prefrontal circuits causally interact during cognitive task performance, moving beyond traditional correlation‐based connectivity. Finally, partial least squares (PLS) linked these neuroimaging biomarkers and clinical cognitive scores (Meidenbauer et al. 2021), establishing an integrative framework. This approach not only identifies early biomarkers of cognitive decline but also helps pinpoint dysfunctional circuits amenable to targeted neuromodulation.

In this study, we investigated directional prefrontal information flow in PD patients across resting and VFT states. First, employing both PLS and paired *t*‐tests, we compared patterns of prefrontal information transfer (quantified via TE) between resting‐state and VFT conditions. Next, we performed PLS analyses to identify task‐state‐specific TE patterns associated with MoCA sub‐scores, revealing distinct EC profiles linked to different cognitive domains. Finally, we correlated VFT‐generated word counts with task‐based TE to pinpoint the prefrontal pathways most predictive of verbal fluency performance. Together, these analyses provide a novel framework for understanding how prefrontal information flow underpins cognitive dysfunction in PD, offering potential biomarkers for early detection and targets for neuromodulation therapies.

## 2 Materials and Methods

### 2.1 Participants

This prospective study enrolled 105 patients with idiopathic PD from the Neurology Outpatient Department of Xinhua Hospital, affiliated with Shanghai Jiao Tong University School of Medicine, between October 2022 and December 2024. Everyone in this sample was right‐hand‐dominant. All participants provided written informed consent after being fully advised of the study's potential benefits and risks. Our study was conducted in accordance with the ethical principles of the Declaration of Helsinki and was approved by the XH‐SJTUSM Ethics Review Committee (Approval Number: XHEC‐C‐2022‐134‐1).

Inclusion Criteria included: (1) aged 60–85 years; (2) clinically diagnosed with PD based on 2015 Clinical Diagnostic Criteria of Movement Disorder Society (MDS) (Postuma et al. 2015); (3) Hoehn‐Yahr (H‐Y) staging I‐III; (4) absence of major neurological or psychiatric comorbidities. Participants were excluded if they had (1) atypical or secondary Parkinsonism; (2) cognitive impairment attributable to stroke, dementia, or other neurological conditions; (3) severe systemic diseases; (4) use of cognition‐affecting drugs within 6 months prior to enrollment; and (5) inability to complete VFT. Due to the poor quality of the fNIRS signal in various measurement channels, 14 participants were excluded based on the following criteria: Participants with >25% of channels (i.e., >13/53 channels) showing coefficient of variation >15% (Piper et al. 2014) during baseline or 20% during task periods. This resulted in a final analyzable sample of 91 participants after quality control.

### 2.2 Clinical Evaluation

Demographic and clinical data were collected for all participants, including age, gender, and years of education. To characterize PD manifestations, we utilized Part II (Activities of Daily Living) and Part III (Motor Examination) of the Unified Parkinson's Disease Rating Scale (UPDRS), while disease progression was assessed using the H‐Y staging scale. Cognitive function was evaluated through MoCA. Additional clinical information included comorbidities and medication history to ensure a thorough characterization of the study cohort. All participants were tested in the ON medication state.

### 2.3 FNIRS Data Collection

**FIGURE 1:**
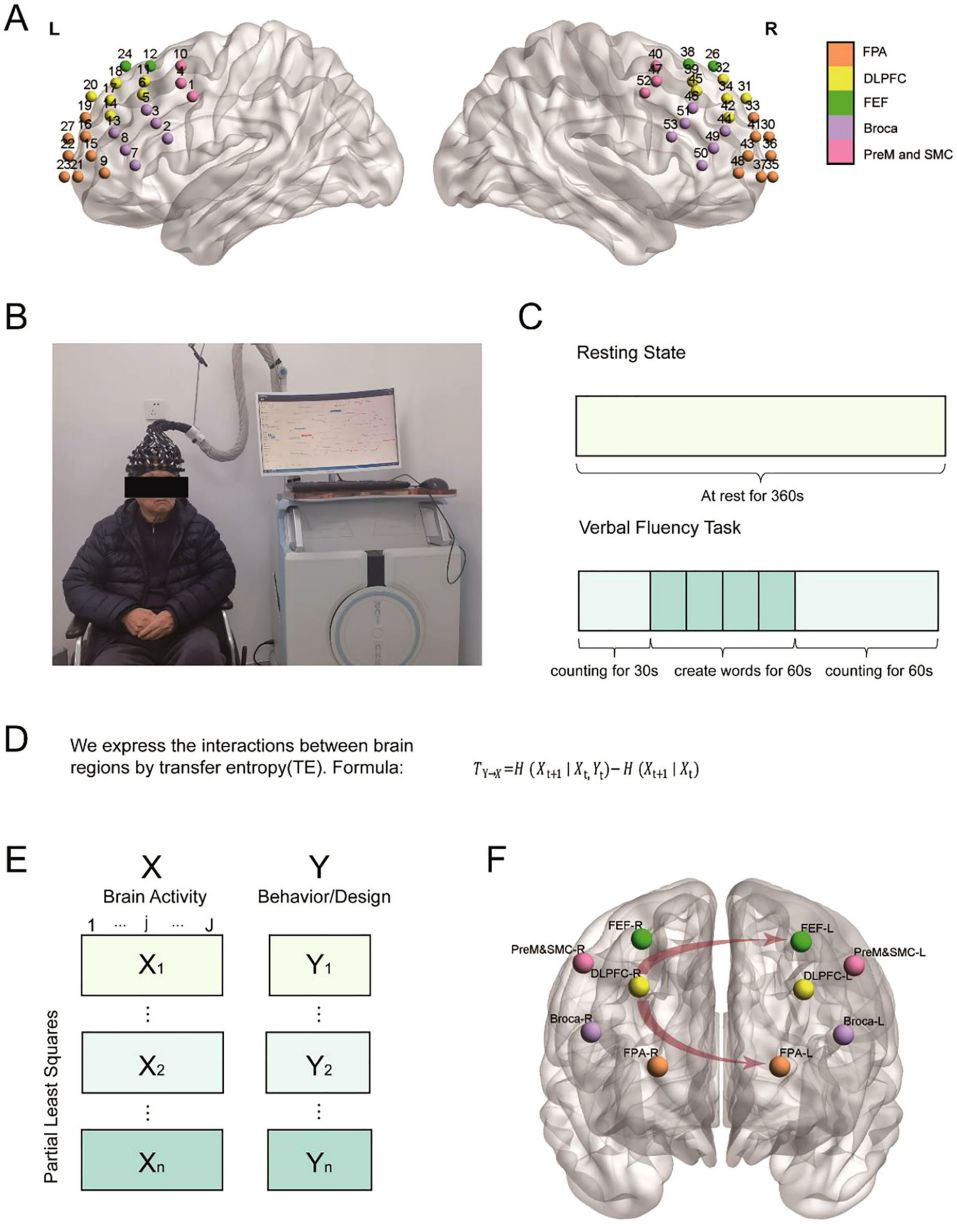
Experimental design and analytical pipeline. (A) fNIRS channels configuration covering prefrontal and temporal cortices. The region is divided into the bilateral pre‐motor and supplementary motor cortex (PreM and SMC), Broca's area (Broca), dorsolateral prefrontal cortex (DLPFC), frontal eye fields (FEF) and frontopolar area (FPA) according to the Brodmann areas. (B) Example of fNIRS recording during a PD patient performing a verbal fluency task. (C) Resting‐state and verbal fluency task procedure. (D) Formular of transfer entropy. (E) Covariance matrix structure integrating brain activity (TE values) with behavior/design for PLS analysis (F) Significant brain‐behavior relationships revealed by PLS.

Participants underwent fNIRS recording in a controlled environment maintained at a comfortable temperature with minimal ambient noise to ensure optimal relaxation. Cerebral hemodynamics were monitored using a 53‐channel BS‐7000 fNIRS system (Wuhan Znion Technology Co., China) with dual‐wavelength near‐infrared spectroscopy (730 nm and 850 nm) at a sampling frequency of 20 Hz, measuring concentration changes of oxygenated (HbO) and deoxygenated hemoglobin (HbR). The optode setup consisted of a flexible headcap incorporating 16 sources and 16 detector optodes arranged in a standardized montage, providing comprehensive coverage of bilateral frontal and temporal lobe regions through 53 measurement channels (Figure 1A). Optode placement followed the international 10–20 system for consistent spatial registration across participants (supplementary material).

The resting‐state protocol and VFT were administered according to our previously published paradigm (Hou et al. 2023). All experimental procedures were conducted by the same trained researcher to ensure consistency. During the 6‐min resting‐state recording, participants were instructed to maintain wakefulness with eyes open while avoiding active cognitive engagement. For the VFT, after a 30‐second counting baseline (1‐15), participants performed four 15‐s trials of Chinese character‐based word generation, followed by a 60‐s counting recovery period (1‐30). Total word production was quantified per trial (Figure 1B,C).

### 2.4 fNIRS Data Preprocessing

All data were processed in MATLAB (2018b) using the Homer2 toolbox V2.8 (https://homer‐fnirs.org/). For resting‐state data, raw signals were converted to optical density (OD) (hmrIntensity2OD), followed by artifact detection (hmrMotionArtifactByChannel) and correction using spline interpolation (hmrMotionCorrectSpline, *p* = 0.99 threshold), and 0.01‐0.2 Hz band‐pass filtering (hmrBandpassFilt) prior to concentration conversion (hmrOD2Conc). Task data underwent identical preprocessing, with block‐averaging (−5 to 115s epochs relative to trial onset) for task responses. Detailed preprocessing procedures are described in our previous study (Tian et al. 2024).

### 2.5 Effective Connectivity

To assess the EC between brain regions, we employed TE (Schreiber 2000), an information‐theoretic measure that quantifies causal influences while accounting for common inputs and temporal dependencies. TE measures the amount of information that the past activity of one brain region (*Y*) provides about the future activity of another region (*X*), beyond what can be predicted from *X*’s own past activity. The formula for calculating TE from *Y* to *X* is as follows:

$$T_{Y\to X}=H\left(X_{t+1}|X_{t},Y_{t}\right)-H\left(X_{t+1}|X_{t}\right)$$

where $T_{Y\to X}$ indicates the TE from Y to X, $H(X_{t+1}|X_{t})$ represents the conditional entropy of X's future given its past, and $H(X_{t+1}|X_{t},Y_{t})$ denotes the conditional entropy when both regions' current states are considered.

The probability distributions required for the entropy calculations were estimated using the histogram binning method, which provides a robust and computationally efficient solution for the data length typical in fNIRS studies. The stability of TE estimates is indeed contingent upon a sufficient number of data points (Y. Wang et al. 2022; Ursino et al. 2020). For our task‐based analysis, the VFT block yielded 60 s of continuous hemodynamic data per participant. Given our fNIRS sampling rate of 20 Hz, this resulted in 1200 data points per channel per task block for the final TE calculation. We computed bidirectional TE for all channel pairs to construct effective connectivity networks, with the asymmetry explicitly capturing directionality in neural information transfer.

### 2.6 Partial Least Squares Analysis

PLS analysis was performed using an established MATLAB toolbox (Krishnan et al. 2011) to identify multivariate relationships between EC patterns and either an experimental design or a set of behavioral measures. PLS analysis procedure comprised (1) constructing a covariance matrix integrating TE‐based connectivity patterns with three sets of conditions: task design matrices, MoCA subscales, and VFT word counts; (2) performing singular value decomposition to extract orthogonal latent variables (LVs); (3) assessing the significance of each LV pattern by 5000 permutation tests (threshold: singular values greater than 95% of the permuted distribution); and (4) identifying the brain salience by 5000 bootstrap tests, defined significant as |bootstrap ratios| > 3.

### 2.7 Statistical Analysis

All other statistical analyses were conducted using SPSS 19 (IBM SPSS Statistics, New York). Continuous variables with normal distribution were presented as mean ± standard deviation (SD), while ordinal variables were expressed as median (interquartile range, IQR). Normality of continuous variables was assessed using the Kolmogorov–Smirnov test, supplemented by visual inspection of Q‐Q plots and histograms. Statistical significance was set at *p* < 0.05 for all tests, with FWE correction for multiple comparisons where applicable.

## 3 Results

### 3.1 Demographic and Clinical Characteristics

The study included 91 PD patients (age range: 60–85 years; mean ± SD: 71.08 ± 5.53 years; 58.2% male). Participants had 3–16 years of education (mean ± SD: 10.50 ± 2.34 years) with the following clinical characteristics: MoCA score 22.15 ± 4.16, UPDRS II score 12.30 ± 7.13, UPDRS III score 28.88 ± 12.74, Hoehn‐Yahr staging (median [IQR]: 2) (Collins and Williams‐Gray 2016; Zhu et al. 2024), and levodopa equivalent daily dose (LEDD) 680.05 ± 355.09 mg/day.

### 3.2 Within‐Individual EC Across Resting and VFT States

**FIGURE 2:**
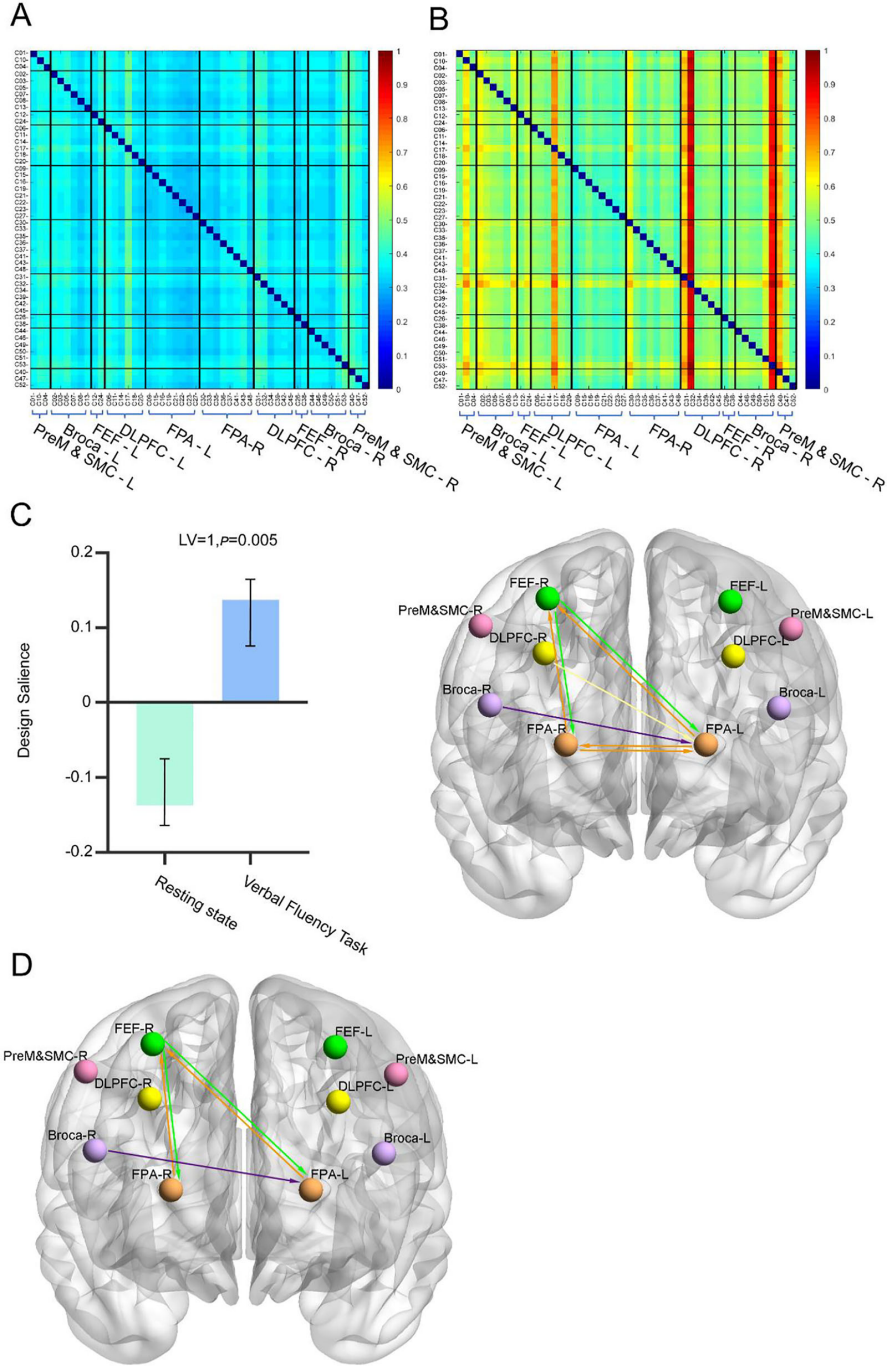
Effective connectivity (EC) changes across cognitive states (resting and verbal fluency task, VFT). TE matrices during resting state (A) and VFT (B). The color at (*x*,*y*) indicates the value of the TE from channel *x* to channel *y*. (C) Design salience of cognitive state on TE and its associated spatial pattern expression from PLS analysis. (D) Significant TE connectivity changes during VFT versus resting state (paired *t*‐test, FEW‐corrected).

Figure 2A,B displayed the EC matrices quantified by TE in PD patients during the resting state and VFT, respectively. Each matrix element (*x*,*y*) indicates the TE value from channel *x* to channel *y*, with warmer colors indicating stronger directional information flow. Compared to the resting state, channel 32 (DLPFC‐R) and 53 (Broca‐R) demonstrated a warmer color during the VFT.

Task‐PLS model revealed a single significant LV (LV1, singular value = 0.19, *p* = 0.0052) capturing state‐dependent EC changes in PD patients. This pattern showed enhanced reciprocal connectivity between the bilateral frontopolar area (FPA) and the right frontal eye fields (FEF‐R), and dominant top‐down information flow from Broca‐R and DLPFC‐R to FPA‐L (Figure 2C).

To further validate these findings, we performed a paired *t*‐test on the TE matrices between resting state and VFT. After family‐wise error (FWE) correction, bidirectional connectivity between FEF‐R and both FPA‐L and FPA‐R, and dominant top‐down flow from Broca‐R to FPA‐L were significantly increased during VFT (Table 1; Figure 2D). Notably, the paired *t*‐test results exhibited strong convergence with the task‐PLS findings, reinforcing the robustness of the observed connectivity patterns.

**TABLE 1:** Task‐induced changes in effective connectivity between prefrontal regions.

Brain region pair	Resting state (TE, mean ± SD)	Verbal fluency task (TE, mean ± SD)	*t*	p*
FPA‐L → FEF‐R	0.33 ± 0.01	0.39 ± 0.09	4.07	1.01×10^−4^
FPA‐R → FEF‐R	0.34 ± 0.09	0.40 ± 0.09	4.08	9.80×10^−5^
FEF‐R → FPA‐L	0.33 ± 0.10	0.39 ± 0.09	4.36	3.50×10^−5^
FEF‐R → FPA‐R	0.35 ± 0.08	0.40 ± 0.09	4.43	4.70×10^−5^
Broca‐R → FPA‐R	0.38 ± 0.09	0.42 ± 0.09	3.71	3.64×10^−4^

Abbreviations: Broca‐R, the right Broca's area; FEF‐R, the right frontal eye fields; FPA‐L, the left frontopolar area; FPA‐R, the right frontopolar area; TE, transfer entropy.

^*^all reported *p* ‐values survive family‐wise error correction at *p* < 0.05.

### 3.3 The Relationship Between Resting‐State TE and MoCA Subscales

**FIGURE 3:**
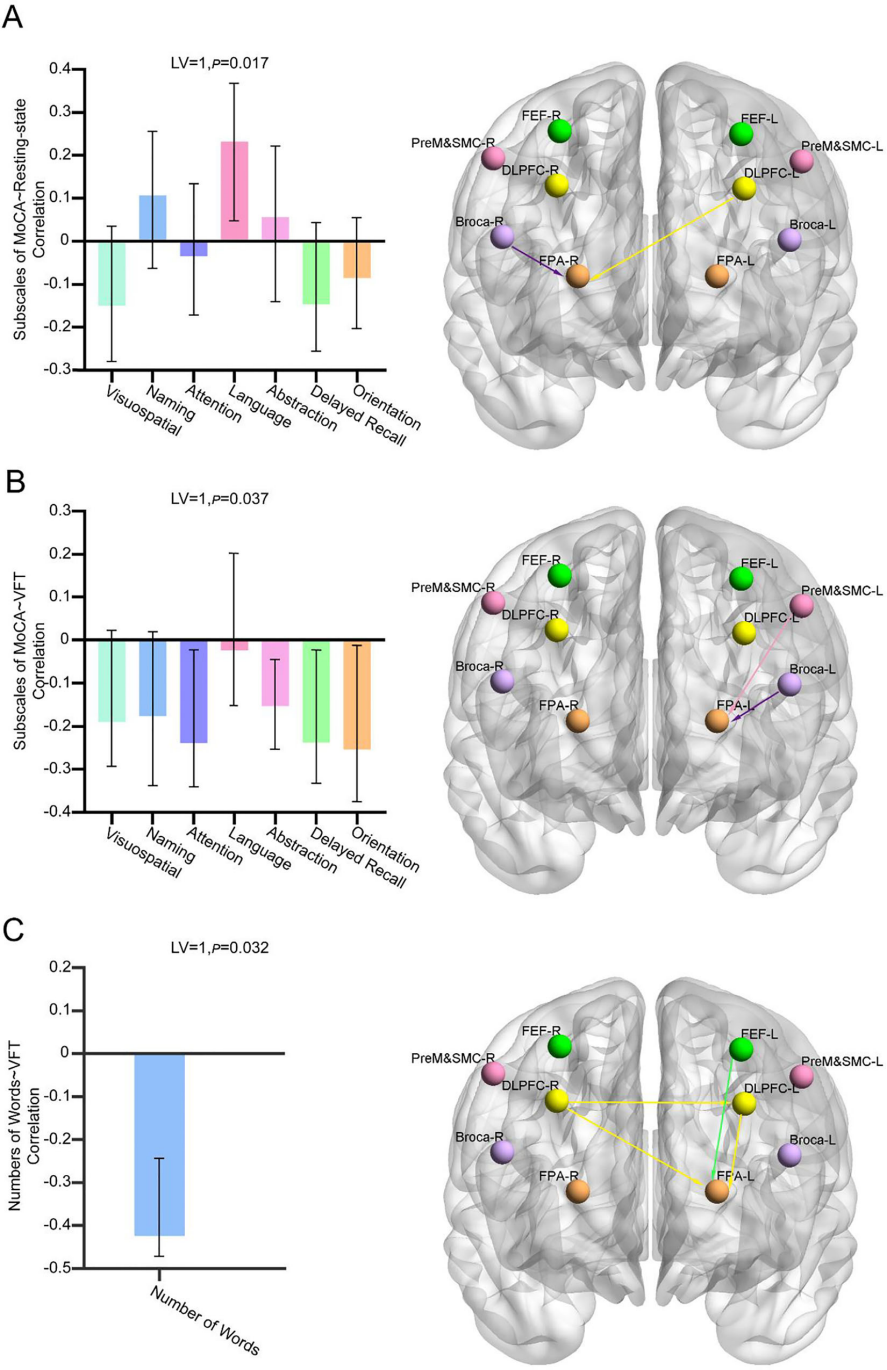
Brain‐behavior relationships revealed by behavioral PLS model. (A) Resting‐state effective connectivity—MoCA subscales correlation. (B) VFT effective connectivity—MoCA subscales correlation. (C) VFT effective connectivity—verbal output correlation.

To investigate how resting‐state effective connectivity relates to cognitive performance, we performed behavioral PLS analysis linking TE pattern with MoCA subscale scores. The result identified one significant LV (LV1, singular value = 1.56, permuted *p* = 0.017) explaining 44.14% of the cross‐block covariance. Higher MoCA language subscores were associated with enhanced information flow Broca‐R→FPA‐R and DLPFC‐L → FPA‐R during resting state (Figure 3A).

### 3.4 The Relationship Between VFT TE and MoCA Subscales

Behavioral PLS analysis of VFT‐related TE pattern revealed one significant LV (LV1, singular value = 2.45, *p* = 0.037), accounting for 61.25% of the cross‐block covariance. This LV showed significant negative correlations with MoCA subscores in attention, abstraction, delayed recall, and orientation. The spatial pattern associated with this effect included enhanced EC from PreM & SMC‐L → FPA‐L and Broca‐L → FPA‐L (Figure 3B).

### 3.5 The Relationship Between VFT EC and the Verbal Output

To investigate how VFT‐based EC relates to VFT performance, we conducted behavioral PLS to link VFT‐based TE patterns with the verbal output. The results showed one significant LV (LV1, singular value = 1.80, permuted p = 0.037), revealing that lower verbal output was associated with enhanced directed connectivity from DLPFC‐R → DLPFC‐L, FEF‐L→FPA‐L, and DLPFC‐L/R → FPA‐L (Figure 3C).

## 4 Discussion

This study investigated the relationship between prefrontal EC patterns and cognitive performance in PD patients. Our study revealed three principal findings: First, EC within the prefrontal network was enhanced during VFT compared to the resting state, suggesting task‐dependent recruitment of prefrontal resources. Second, stronger top‐down connectivity targeting the FPA‐R during resting state correlated with better language performance on the MoCA, implicating FPA‐R in baseline language network efficiency. Third, and most critically, increased top‐down connectivity to the FPA‐L during VFT was associated with poorer performance across multiple MoCA subdomains (attention, abstraction, delayed recall, and orientation) and reduced verbal output, potentially reflecting compensatory overactivation in response to cognitive decline.

Our study demonstrated the critical role of the FPA in mediating cognitive performance in PD. Task PLS revealed significantly enhanced information transfer between FPA‐L, FPA‐R, and FEF‐R from resting state to VFT, accompanied by increased top‐down inputs from Broca‐R and DLPFC‐R to FPA‐L, suggesting their coordinated involvement in task execution. These findings align with established evidence of FPA's cognitive functions—from Miyamoto K et al.’s EEG studies showing FPA activation during higher‐order cognition in humans and primates (Miyamoto et al. 2023) to clinical reports of FPA‐lesioned patients exhibiting preserved basic intelligence but impaired complex task performance (Mansouri et al. 2015) —collectively underscoring FPA‐L as an integrative hub.

This FPA‐centered network operates in concert with other specialized prefrontal regions. The FEF cortex contributes crucially to memory indexing functions while maintaining reciprocal connections with the FPA through striatal‐frontal projections (Rolls et al. 2022). Complementing this network, the DLPFC not only facilitates task‐switching flexibility through its regulation of the frontopolar cortex (Hogeveen et al. 2022) but also supports essential cognitive operations, including attentional control, working memory integration, and sensory information processing (Huang et al. 2022). Importantly, fNIRS studies have demonstrated age‐related strengthening of functional connectivity between Broca's area and the FPA, along with enhanced interhemispheric integration during task performance (Laguë‐Beauvais et al. 2013). Within this integrated network, our results demonstrated that PD patients recruit FPA‐L, FPA‐R, FEF‐R, Broca‐R, and DLPFC‐R in a coordinated manner during VFT compared to the resting state, with FPA‐L emerging as a pivotal information integration hub for integrating these inputs to support task execution.

Our findings demonstrated that the FPA receives extensive inputs from other prefrontal regions, potentially representing a compensatory mechanism for cognitive decline in PD. Behavioral PLS analysis revealed two EC patterns: (1) Resting‐state connectivity from Broca‐R/DLPFC‐L → FPA‐R positively correlated with language subscales, while (2) during VFT, increased information flow from PreM&SMC‐L/Broca‐L →FPA‐L negatively correlated with attention, abstraction, delayed recall, and orientation subscales. Notably, poorer VFT performance (fewer words output) was associated with enhanced DLPFC‐R/FEF‐L → FPA‐L connectivity. Within the framework of PD‐related dopaminergic denervation and frontostriatal dysfunction, these results suggest two non‐exclusive possibilities: (1) FPA‐L mediated compensatory scaffolding, as proposed by the Scaffolding theory of Aging and Cognition (Oosterhuis et al. 2023) albeit in the context of PD‐specific dopaminergic depletion and striatal decoupling, or (2) dopamine depletion‐induced maladaptive hyperconnectivity that further disrupts cognitive processing (Rodriguez‐Rojas et al. 2022) While Broca's area and DLPFC normally support language functions (Metzger et al. 2016, Ford et al. 2010), their increased EC to FPA‐L may reflect either compensatory reorganization or dysfunctional decoupling from striatal inputs. Notably, a U‐shaped relationship between FPA‐L connectivity and cognitive function might exist, where initial increases are compensatory but later exacerbations become detrimental—a pattern observed in Alzheimer's disease networks (Schultz et al. 2017). This interpretation aligns with established FPA roles in higher‐order cognition (Ainsworth et al. 2022) and age‐related frontal changes (J. Wang et al. 2020). The FPA‐L's integrative capacity—receiving inputs from motor (PreM&SMC), language (Broca), and executive (DLPFC/FEF) regions—positions it as a critical hub for maintaining cognitive function amid neurodegenerative progression. Future intervention studies (e.g., FPA‐L‐targeted neuromodulation) could test these causality mechanisms.

This study demonstrated three key methodological strengths: First, we pioneered the integration of TE with PLS analysis in fNIRS research, overcoming limitations of traditional functional connectivity approaches by not only capturing prefrontal hemodynamic changes in PD patients but also revealing directional information transfer between brain regions. Second, we established a multimodal data integration framework that incorporates both resting‐state and VFT EC patterns, multi‐domain MoCA scores, and behavioral measures (verbal output) into a unified model, enabling precise correlation between neural activity and cognitive performance. Third, through cross‐validation with conventional *t*‐test results, we confirmed the reliability of our identified neural activation patterns while discovering the critical role of the FPA‐L as an information integration hub, providing objective neuroimaging biomarkers for early detection of cognitive impairment in PD.

While our study revealed task‐dependent prefrontal effective connectivity patterns linked to cognitive performance in PD, certain limitations should be acknowledged. First, the absence of short channels in our fNIRS setup limited our ability to fully separate superficial hemodynamic artifacts from cortical signals, though task‐baseline contrasts helped mitigate this issue. Future studies should incorporate advanced signal processing techniques (e.g., PCA‐based regression) to improve signal specificity (Zhang et al. 2021). Second, while fNIRS provided dynamic prefrontal assessment, its restricted spatial coverage precluded examination of deeper or posterior regions potentially involved in cognitive networks. Third, although conducting all assessments in the ‘ON’ medication state standardized testing conditions and minimized motor confounds, this design precluded assessment of medication effects on connectivity. The potential modulation by dopaminergic therapy warrants investigation in future ON/OFF comparisons or through LEDD covariance analyses. Fourth, while TE provided directional connectivity estimates, it remained a statistical measure and cannot establish direct neural causality—future studies combining neuromodulation techniques (e.g., TMS) could further validate these findings. Fifth, the behavioral measures used (MoCA subscales and VFT performance) may lack sensitivity for detecting subtle cognitive decline in early‐stage PD; incorporating more comprehensive neuropsychological tests or longitudinal designs could enhance detection accuracy. Finally, the absence of a healthy control group limits our ability to disentangle PD‐specific connectivity alterations from general age‐related changes, highlighting the need for comparative studies in future research.

## 5 Conclusion

In conclusion, our study establishes the FPA‐L as a critical neural hub whose functional connectivity patterns (integrating inputs from PreM&SMC‐L, Broca‐L, DLPFC, and FEF‐L) are strongly associated with cognitive performance in PD. The observed negative correlation between FPA‐L connectivity strength and cognitive function levels may suggest either compensatory network reorganization or maladaptive hyperconnectivity in response to neurodegenerative processes. Importantly, the FPA‐L's dual role as both a biomarker for cognitive dysfunction progression and a potential neuromodulation target provides a translational bridge between mechanistic understanding and clinical applications. These findings advance our understanding of PD‐related cognitive decline pathogenesis while offering novel avenues for developing targeted interventions.

## Author Contributions

**Xinxin Chen**: writing – original draft, investigation, data curation. **Bingjie Tian**: data curation, investigation. **Bingjie Ma**: data curation, investigation. **Shizheng Zhang**: writing – review & editing. **Xiangdong Huang**: visualization. **Jianping Huang**: visualization. **Zhenguo Liu**: supervision, resources, conceptualization. **Miaomiao Hou**: methodology, software, formal analysis.

## Conflicts of Interest

The authors declare no conflicts of interest.

## Peer Review

The peer review history for this article is available at https://publons.com/publon/10.1002/brb3.70915

## Supporting information




**Supplementary Material**: brb370915‐sup‐0001‐TableS1.docx

## Data Availability

The datasets generated and/or analyzed during the current study are available from the corresponding author upon reasonable request.
