# Supplementary material for: Effective Functional Pattern Between Prefrontal fNIRS Activity and Cognitive Performance in Patients With Parkinson's Disease: A Data‐Driven PLS Approach
Source: Brain Behav. 2025 Sep 25;15(9):e70915. doi: 10.1002/brb3.70915 (PMC12463692; doi:10.1002/brb3.70915)
Supplement: Supplementary file 1 — Supplementary Material: brb370915‐sup‐0001‐TableS1.docx [file BRB3-15-e70915-s001.docx]

**Supplementary table 1** MNI coordinates for all channels and corresponding Brodmann area mappings

| Channel | MNI coordinates | | | Region Label according to Brodmann area |
| --- | --- | --- | --- | --- |
|  | x | y | z |  |
| 1 | -57 | 6 | 40 | PreM & SMC-L |
| 2 | -60 | 19 | 18 | Broca-L |
| 3 | -56 | 25 | 26 | Broca-L |
| 4 | -50 | 12 | 47 | PreM & SMC-L |
| 5 | -51 | 30 | 33 | Broca-L |
| 6 | -45 | 32 | 41 | DLPFC-L |
| 7 | -55 | 36 | 4 | Broca-L |
| 8 | -52 | 41 | 12 | Broca-L |
| 9 | -47 | 52 | 0 | FPA-L |
| 10 | -45 | 12 | 56 | PreM & SMC-L |
| 11 | -39 | 31 | 48 | DLPFC-L |
| 12 | -30 | 28 | 56 | FEF-L |
| 13 | -46 | 47 | 21 | Broca-L |
| 14 | -40 | 49 | 30 | DLPFC-L |
| 15 | -40 | 59 | 9 | FPA-L |
| 16 | -32 | 62 | 19 | FPA-L |
| 17 | -31 | 49 | 38 | DLPFC-L |
| 18 | -20 | 46 | 47 | DLPFC-L |
| 19 | -21 | 62 | 29 | FPA-L |
| 20 | -12 | 59 | 40 | DLPFC-L |
| 21 | -33 | 66 | -2 | FPA-L |
| 22 | -22 | 71 | 9 | FPA-L |
| 23 | -13 | 73 | -2 | FPA-L |
| 24 | -11 | 41 | 56 | FEF-L |
| 25 | 0 | 51 | 45 | DLPFC |
| 26 | 12 | 42 | 56 | FEF-R |
| 27 | -13 | 71 | 18 | FPA-L |
| 28 | 1 | 63 | 28 | FPA |
| 29 | 0 | 68 | 8 | FPA |
| 30 | 13 | 71 | 19 | FPA-R |
| 31 | 12 | 59 | 39 | DLPFC-R |
| 32 | 21 | 47 | 49 | DLPFC-R |
| 33 | 22 | 63 | 29 | FPA-R |
| 34 | 31 | 49 | 39 | DLPFC-R |
| 35 | 13 | 73 | -2 | FPA-R |
| 36 | 24 | 72 | 9 | FPA-R |
| 37 | 34 | 67 | -2 | FPA-R |
| 38 | 30 | 29 | 57 | FEF-R |
| 39 | 40 | 31 | 48 | DLPFC-R |
| 40 | 47 | 12 | 56 | PreM & SMC-R |
| 41 | 33 | 64 | 19 | FPA-R |
| 42 | 41 | 50 | 29 | Broca-R |
| 43 | 43 | 60 | 9 | FPA-R |
| 44 | 49 | 48 | 22 | Broca-R |
| 45 | 46 | 33 | 43 | DLPFC-R |
| 46 | 53 | 31 | 34 | Broca-R |
| 47 | 52 | 12 | 48 | PreM & SMC-R |
| 48 | 48 | 55 | 0 | FPA-R |
| 49 | 54 | 42 | 13 | Broca-R |
| 50 | 58 | 36 | 4 | Broca-R |
| 51 | 58 | 27 | 26 | Broca-R |
| 52 | 60 | 6 | 42 | PreM & SMC-R |
| 53 | 62 | 20 | 19 | Broca-R |

Abbreviation: PreM &SMC-L, the left pre-motor and supplementary motor cortex; PreM &SMC-R, the right pre-motor and supplementary motor cortex; DLPFC-L, the left dorsolateral prefrontal cortex; DLPFC-R, the right dorsolateral prefrontal cortex; FPA-L, the left frontopolar area; FPA-R, the right frontopolar area; FEF-L, the left frontal eye fields; FEF-R, the right frontal eye fields; Broca-L, the left Broca's area; Broca-R, the right Broca's area.
